# The antibiotic bead pouch – a useful technique for temporary soft tissue coverage, infection prevention and therapy in trauma surgery

**DOI:** 10.5194/jbji-8-165-2023

**Published:** 2023-06-21

**Authors:** Markus Rupp, Nike Walter, Dominik Szymski, Christian Taeger, Martin Franz Langer, Volker Alt

**Affiliations:** 1 Department of Trauma Surgery, University Hospital Regensburg, Franz-Josef-Strauss-Allee 11, 93053 Regensburg, Germany; 2 Plastische Chirurgie & Ästhetik an der Isar, Widenmayerstraße 16, 80538 Munich, Germany; 3 Department of Trauma, Hand and Reconstructive Surgery, Waldeyerstrasse 1, 48149 Muenster, Germany

## Abstract

Soft tissue defects resulting from trauma and musculoskeletal infections can complicate surgical treatment. Appropriate temporary coverage of these defects is essential to achieve the best outcomes for necessary plastic soft tissue defect reconstruction. The antibiotic bead pouch technique is a reasonable surgical approach for managing temporary soft tissue defects following adequate surgical debridement. This technique involves the use of small diameter antibiotic-loaded bone cement beads to fill the dead space created by debridement. By applying antibiotics to the bone cement and covering the beads with an artificial skin graft, high local dosages of antibiotics can be achieved, resulting in the creation of a sterile wound that offers the best starting position for soft tissue and bone defect reconstruction.

This narrative review describes the rationale for using this technique, including its advantages and disadvantages, as well as pearls and pitfalls associated with its use in daily practice. In addition, the article provides a comprehensive overview of the literature that has been published since the technique was introduced in surgical practice.

## 1 Introduction

Musculoskeletal injuries can lead to soft tissue defects, particularly in open fractures classified by Gustilo–Anderson as type IIIb or type IIIc, both of which require plastic surgical soft tissue coverage. In type IIIc fractures, nerve or vessel injuries are also present (Gustilo and Anderson, 1976). Soft tissue damage is a common problem in bone infection, with the extent of soft tissue damage predisposing to the development of fracture-related infections (FRIs). The incidence of FRIs varies depending on the anatomic region, with rates ranging from 1.8 % in Gustilo type I fractures to 42.9 % in Gustilo type IIIb tibial fractures (Ktistakis et al., 2014; Papakostidis et al., 2011). When soft tissue defects complicate open fracture management, best possible prophylaxis is required to avoid FRI. When already established FRI is present, the goals of therapy are infection eradication and soft tissue and bone defect reconstruction. In cases where FRI has already developed, the goals of therapy are to eradicate infection, reconstruct soft tissue and bone defects, and minimize the socioeconomic and treatment burden for patients (Walter et al., 2021a, b).

Chronic osteomyelitis is characterized by infected, dead bone within a compromised soft tissue envelope, according to George Cierny, who paved the way for better understanding and treatment of this condition. Treatment of chronic osteomyelitis and FRI typically involves adequate bony debridement to remove necrotic bone tissues, as well as resection of compromised soft tissues, followed by plastic surgical flap coverage and antibiotic therapy (Lowenberg et al., 2019; Metsemakers et al., 2019).

There are several therapy options available for temporary soft tissue coverage. Sterile dressings with gauze or towels are a simple and inexpensive option but require regular changes and can be uncomfortable for the patient. Negative pressure wound therapy (NPWT), also known as vacuum-assisted wound closure, has been widely used in surgical therapy since the early 1990s, particularly in trauma and orthopedic surgery. NPWT improves local blood supply and simultaneously suctions wound exudate, promoting wound healing Low-quality data indicate that NPWT is beneficial for wound closure and can reduce hospital-stay time (Zens et al., 2020). However, high bacterial loads have been found in foams removed from NPWT (Yusuf et al., 2013). The clinical relevance of this observation is not clear, but it may be one reason why definite soft tissue closure is recommended within 7 d (Haidari et al., 2021; Sweere et al., 2022).

**Figure 1:**
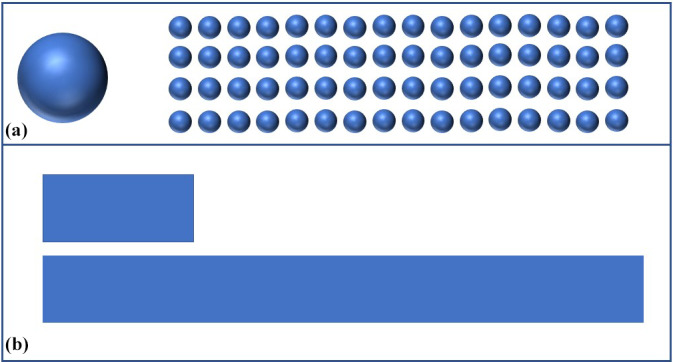
**(a)** Exemplary comparison of the same volumes in one sphere compared to many smaller spheres that make up the same volume in total. For example, 40 g of PMMA bone cement can be used to form one sphere with a radius of about 2 cm or about 64 spheres with a radius of 0.5 cm. The surface area of the 64 smaller spheres is over 4 times **(b)** the surface area of the single sphere. The smaller the spheres can be formed during the time available for cement curing, the greater the surface area and thus the elution of antibiotics from the PMMA bone cement.

The use of an antibiotic bead pouch is a viable option for temporary soft tissue coverage. Adequate temporary soft tissue coverage is essential for the successful treatment of traumatic or posttraumatic soft tissue defects. A healthy soft tissue envelope ensures proper blood supply to the area of bone healing and protects the healing tissues from bacterial contamination. Furthermore, sufficient blood supply provides ample perfusion of immune cells and systemically administered antibiotics (Gosain et al., 1990; Moriarty et al., 2022). In contaminated wounds after an open fracture or established FRIs, achieving a healthy wound that allows desired healing is crucial. Thorough debridement and irrigation reduce the bacterial load by removing necrotic soft tissue. The reduction of the pathogen burden and viable, well-vascularized tissue is essential to enable the host immune system to eradicate wound infection-causing pathogens (Lenarz et al., 2010; Robinson et al., 1989). The debridement of bone and soft tissue usually results in a tissue defect, which is usually filled with a hematoma. This provides nutrients for bacterial growth and can be considered a favorable environment for bacterial growth. To avoid bacterial growth by reducing the volume of dead space and filling it with antibiotics, the management of dead space has become a cornerstone of septic surgery principles. Different biomaterials are available for dead space management, and PMMA beads have been used as dead space fillers for more than 40 years (Wahlig et al., 1978). Calcium sulfate is another biomaterial that enables local antibiotic application and has been historically used as a bone void filler. It is also being studied for dead space management of soft tissues. A concern with its use in soft tissues is the induction of heterotopic ossification, which was not confirmed in an animal model (Oliver et al., 2018). Meanwhile, other viable options for local application of antibiotics exists in surgical practice. In recent years, application of pure antibiotic powder such as vancomycin has been proven to reduce infection risk in fracture care (Marchand et al., 2023; Wang et al., 2023). In addition, collagen sponges soaked with antibiotics are another option to apply antibiotics to the surgical site (Chaudhary et al., 2011). Both options, collagen sponges and powder, have their limitations for dead space management and wounds that require temporary soft tissue coverage. Sustained release is not provided when powder is administered locally. Both antibiotic powder and sponges are not able to fill the dead space as adequately as biomaterials with solid consistency, such as PMMA bone cements or calcium sulfate beads. However, the major drawback of using calcium sulfate beads in an antibiotic bead pouch is its cost. PMMA bone cements are by far less expensive and therefore a more feasible option. Local antibiotic carriers allow for very high local antibiotic concentrations that do not result in significant negative systemic side effects. Due to the high antibiotic concentrations, biofilms and bacteria initially tested resistant in conventional culture diagnostics can still be eradicated (Malchau et al., 2021). The preparation of small antibiotic-loaded bone cement beads is particularly useful, since the increased surface area can significantly increase the antibiotic release from the polymethyl methacrylate (PMMA) cement (Fig. 1a and b).

During the surgical procedure, proper wound irrigation should be performed after adequate debridement (Investigators et al., 2015). Additionally, systemic antibiotic prophylaxis or therapy, as well as the application of local antibiotic carriers, is an established standard and should be based on the form of therapy (prophylaxis or therapy) and the soft tissue situation. For open fractures with extensive soft tissue involvement (Gustilo type III), 3 d of systemic antibiotic therapy should be administered, covering both gram-positive and gram-negative bacteria. Recommended antibiotic combinations for infection prophylaxis in open type III Gustilo fractures include ampicillin and sulbactam, piperacillin, and tazobactam, as well as ceftriaxone and vancomycin (Garner et al., 2020; Omar et al., 2021). Typically, 6 to 12 weeks of systemic targeted antibiotic therapy is required for bone infections (Depypere et al., 2020). Considering this orchestrated approach, the antibiotic bead pouch is a viable surgical method for temporary soft tissue coverage, adequate dead space management, and reduction of bacterial load to achieve the long-term therapeutic goal of limb reconstruction and restoration of function, which will be outlined in the following sections.

**Table 1:** Advantages and disadvantages of using antibiotic bead pouches for temporary soft tissue coverage.

Advantages	Disadvantages
High antibiotic concentrations can significantly reduce bacterial counts, preventing infections and adequately treating established infections. Antibiotics have been reported to elute from PMMA bone cement for up to 28 d (Slane et al., 2018). The materials required to create an antibiotic bead pouch are readily available in most surgical orthopedic and trauma facilities, and they are also cost effective In addition to broad empirical antibiotic treatment, targeted antibiotic therapy can be initiated based on the existing resistogram when changing the antibiotic bead pouch. Local antifungal therapy is also an option. The number of subsequent interventions can be reduced, thereby avoiding pathogen changes that often occur after multiple debridement stages (Rupp et al., 2022, 2020). The use of an antibiotic bead pouch does not affect temporary fracture stabilization by internal or external fixation. The nursing effort required until definitive soft tissue reconstruction is limited to standard dressing changes. There is no need for elaborate materials such as electrically operated pump systems, as is the case with NPWT therapy. Medical and nursing staff are significantly less stressed in terms of time and effort, as leaks and other complications associated with NPWT therapy are avoided with the use of an antibiotic bead pouch.	Delaying plastic coverage for 7–10 d may require another change of the antibiotic bead pouch. For very extensive or circular soft tissue defects such as degloving injuries, the use of an antibiotic bead pouch may not be technically feasible. In such cases, temporary soft tissue coverage using NPWT therapy could be an option to effectively prepare the wound bed.

## 2 Indications and contraindications

In the initial treatment of open fractures (Gustilo type IIIb and IIIc), the antibiotic bead pouch technique is a suitable therapy option for infection prevention. If primary wound closure is achievable (Gustilo type I, II, and IIIa), a local antibiotic carrier should be applied to the fracture site, as it has been shown to reduce reinfection and osteomyelitis rates (Morgenstern et al., 2018). In such a scenario, an antibiotic bead pouch is usually not necessary, and primary wound closure is the surgical therapy of choice. However, in cases of extensive soft tissue damage (Gustilo type IIIb and IIIc), temporary soft tissue coverage is required, and the antibiotic bead pouch is a useful means to achieve temporary soft tissue coverage for infection prevention. If surgical debridement of already established infections such as FRIs and chronic osteomyelitis results in a soft tissue defect with exposed bone, the antibiotic bead pouch is a suitable therapy option to fill the dead space around the bone, creating an optimal situation for plastic coverage. If coverage of the bone by muscle tissue is necessary, NPWT therapy may be useful in conditioning the wound bed early for mesh graft or other plastic procedures. In any case, plastic coverage should be aimed for within 7 d (Ostermann et al., 1994; Pincus et al., 2019).

In the author's experience, pure soft tissue defects without bony involvement do not benefit from antibiotic bead pouch treatment. In case of isolated soft tissue defects, there is no bony dead space that needs to be antibiotically shielded for subsequent bone reconstruction. NPWT therapy can usually condition the soft tissue for further plastic reconstructive procedures with good granulation tendency.

**Table 2:** Pearls for clinical management and surgical preparation.

Patient information
Gather information about necessary follow-up operations with bone defect reconstruction or osteosynthesis as well as plastic coverage. Gather information about allergic reactions to local antibiotics and components of PMMA bone cement.
Surgical preparation
Positioning of the patient according to the anatomical region and the surgical access route. *Preoperative skin antisepsis and systemic antibiotic infection therapy or prophylaxis (Stanton, 2021).* In case of infection prophylaxis, withholding perioperative antibiotics does not result in better diagnostic yield and should therefore be administered 60 min before skin incision (Bratzler and Houck, 2004; Mielke and Hansis, 2018).
Instrumentation
Polymethyl methacrylate (PMMA) bone cement powder and liquid. Mixing vessel, spatula, and pestle if necessary. Antibiotic powder (in the case of empirical antibiotic administration, vancomycin for the gram-positive germ spectrum and gentamicin or meropenem for the gram-negative germ spectrum (Rupp et al., 2021); otherwise, targeted antibiotic therapy, taking into account the heat stability of the antibiotic, since significant heat is generated during the hardening of the cement as part of the exothermic reaction). Synthetic wound dressing such as Epigard^®^ or SYSpurderm^®^.
Producing the beads
Mix the bone cement powder with the antibiotic powder. Add the bone cement liquid antibiotic powder to the bone cement. A curing time of 2 min is recommended. Small beads should be formed afterwards. Producing a bead chain by putting the beads on a non-resorbable suture is also a feasible option. Complete curing of the beads should be awaited prior to placement of the beads into the situs.

## 3 Surgical technique

**Figure 2:**
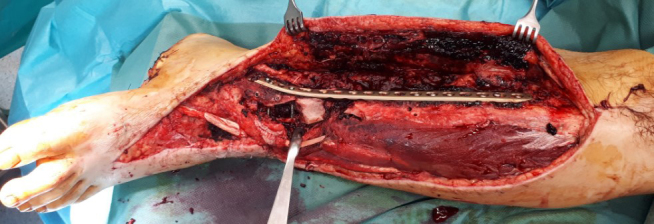
Intraoperative finding after surgical debridement of necrotic soft tissues and bone after internal fixation of the pilon fracture with a distal tibia locking plate (Depuy-Synthes; Zuchwil, Switzerland).

Thorough surgical debridement should be performed in cases like that of the 38-year-old patient who suffered a Gustilo type IIIb distal tibial fracture (Fig. 2) as a result of a motorcycle accident.

**Figure 3:**
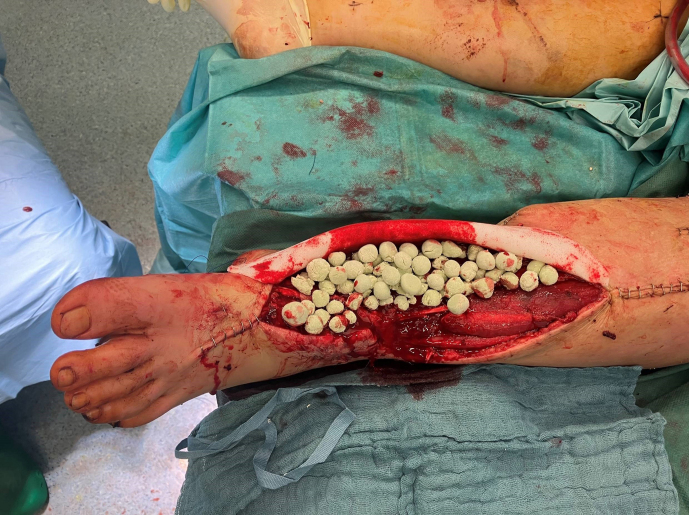
Antibiotic-loaded bone cement beads are applied to the wound. Artificial skin graft (in this case, Epigard^®^; Biovision Biomaterial, Ilmenau, Germany) covers the wound for temporary soft tissue closure.

**Figure 4:**
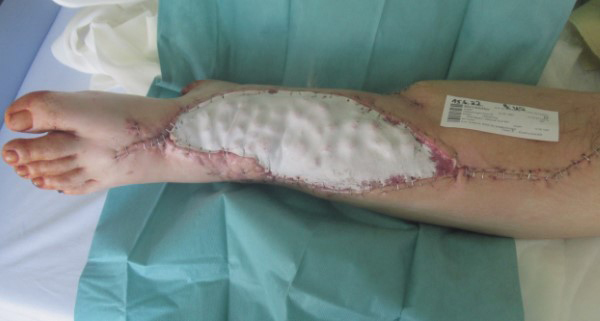
The Epigard^®^ fixed with staples securely seals the antibiotic-loaded PMMA beads and thus guarantees a sterile environment until plastic soft tissue coverage.

The complete resection of non-viable bone and soft tissue was necessary, followed by the stabilization of the fracture using external or internal osteosynthesis. In this particular case, thorough debridement was achieved through extensive wound irrigation with saline via jet lavage, using a total of 9 L of saline, and treatment of bone and soft tissues with Granudacyn wound irrigation solution (Mölnlycke Health Care GmbH, Düsseldorf, Germany). Temporary soft tissue coverage was required until definitive plastic restoration could be performed through free flap surgery. For this purpose, an antibiotic bead pouch was utilized. At the instrument table, the surgical team mixed 40 g of PMMA cement (Palacos R^®^; Heraeus, Wehrheim, Germany) with 2 g each of vancomycin and meropenem powder. After a curing time of 2 min, the mass was formed into beads with the smallest possible diameter and placed into the situs. The antibiotic-loaded PMMA cement beads were covered, and the wound was sealed with a temporary skin substitute (Fig. 3). Since the artificial skin does not cover viable soft tissues directly, it does not require regular changing and can remain in place until definite plastic coverage (Fig. 4). In this case, the bead pouch remained in place for 3 weeks until free flap coverage was performed by a microsurgeon.

## 4 Special features of the surgical technique

**Figure 5:**
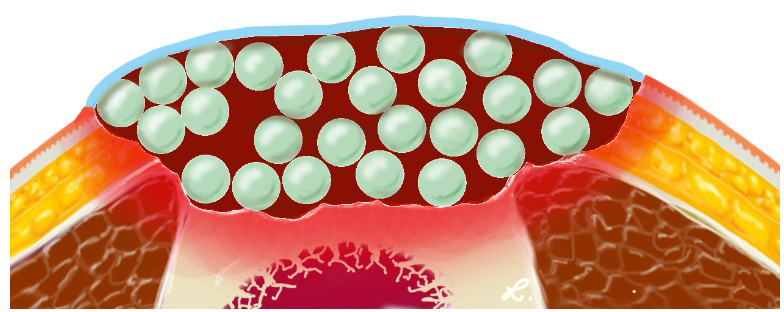
Illustration of the antibiotic beads (light green) providing high local concentrations of antibiotics in the hematoma (dark red). The hematoma and the beads are covered by an artificial skin graft (light blue).

The technique of creating an antibiotic bead pouch involves the use of a synthetic skin substitute and the fabrication of antibiotic-laden beads. While previous publications have described covering bone cement balls with opsite film (Ostermann et al., 1989) or using commercially available gentamicin-loaded chains (Septopal^®^; Zimmer Biomet, Vienna, Austria) to create an antibiotic bead pouch (Bowyer, 1993), the reasons for deviating from these techniques in the present case are worth explaining for better understanding of its practicability. Mixing different antibiotics provides several advantages. First, it allows for optimal empiric local antibiotic therapy, such as vancomycin and meropenem or vancomycin and gentamicin. When selecting local antibiotics for empirical therapy in PMMA, it is necessary to consider the suspected pathogens causing the infection, as well as the technical feasibility of mixing antibiotics with bone cement. For this purpose, antibiotics need to be heat resistant and available in powder form. Our previous investigations have identified several suitable antibiotic combination therapy options for empirical therapy. Among these, vancomycin and meropenem have proven to be useful and practical due to their physical properties during preparation with PMMA cement (Rupp et al., 2021). Additionally, targeted antibiotic therapy can be performed using this technique. Although antibiotic combination preparations for bone cements are available on the market, they can be quite expensive and are mainly reserved for revision arthroplasty cases where the aim is to prevent recurrence of infection while ensuring optimum stability of the cement and fixation of revision implants that will remain in the patient for life. In contrast, in the case of PMMA bone cement spacers, stability is not of decisive importance for the antibiotic bead pouch. The PMMA bone cement primarily functions as an antibiotic carrier and achieves a sterile wound, releasing high concentrations of antibiotics to the hematoma around the beads (Fig. 5). The amount of antibiotics used in the described technique is based on the current recommendation of a maximum admixture amount of 10 % (2 g vancomycin and 2 g meropenem is 4 g antibiotics per 40 g PMMA bone cement) (Kühn et al., 2017).

In addition to the benefits of using high dosages of antibiotics, the combination of two antibiotics also improves the release of antibiotics from the bone cement. This is important because only a maximum of 15 % of the antibiotics in PMMA bone cement is released from the surface, with the majority being released within the first 48 h after implantation (Geurts and Walenkamp, 2017; Kühn et al., 2017). The use of synthetic skin substitutes is also preferred, as they can be easily fixed into the wound edges and sealed, allowing for effortless daily dressing changes. Using film to seal the antibiotic beads may result in detachment from the skin and increased wound secretion, and changing the film may also contaminate the situs. Changing the antibiotic beads every 48–72 h is not necessary when synthetic skin substitutes are used, and, with thorough debridement, the antibiotic bead pouch can be left in place until plastic reconstruction. The authors do not recommend staged revisions every 48–72 h, as this can result in pathogen changes, unnecessary consumption of surgical resources, and added stress for the patient (Rupp et al., 2022, 2020).

## 5 Errors, pitfalls, and complications

Larger defects treated with the antibiotic bead pouch involve the risk of extensive blood loss. Thorough debridement must nevertheless be performed because leaving infected and necrotic tissue in place is contrary to the actual therapeutic goal. However, clinical experience shows that if the clinical findings are not certain, a multi-stage procedure may be useful to wait for demarcation of the vital from the avital tissue. In this way, unnecessarily wide, soft tissue resections, which are then again difficult to reconstruct, can be avoided. During debridement, subtle hemostasis is mandatory to prevent postoperative bleeding, and if bleeding occurs from the bone, particularly from the medullary canal, it can be closed with PMMA bone cement. Care should be taken not to apply the cement when it is still very liquid, as it may be inserted deep into the medullary canal and the cancellous bone, making removal more difficult in a later surgical procedure. To avoid small antibiotic beads from disappearing unintentionally into the medullary canal, the authors recommend sealing the medullary canal with PMMA bone cement. Removal of a PMMA bead can be tedious in the course and is avoidable. If one wishes to fill the medullary canal with local antibiotic carriers, one can either use absorbable antibiotic carriers such as antibiotic fleece or apply antibiotic carriers containing calcium sulfate. Optional antibiotic bone-cement-coated rods can also fill and additionally stabilize the affected bone (Ismat et al., 2021) Commercially available antibiotic bead chains can also be inserted into the medullary canal but are often difficult to remove in the course.

The PMMA beads made in the wound bed should be as small as possible, with a diameter of 0.5–1 cm per bead being practical. The number of implanted beads should be noted in the surgical report and counterchecked in the subsequent operation when the beads are removed. To facilitate removal in the follow-up surgery, an antibiotic bead chain can be made with a nonabsorbable suture. The synthetic skin substitute used in the treatment should be removed completely during the follow-up surgery, and moistening is usually not necessary. The PMMA bone cement beads and the hematoma surrounding them prevent larger granulation and ingrowth of the soft tissue into the synthetic material.

## 6 Results – the antibiotic bead pouch in literature

A literature search conducted on http://PubMed.gov on 22 March 2023 yielded 24 publications related to “antibiotic bead pouch”. Antibiotic-loaded bone cement was first introduced in arthroplasty by Wilhelm Buchholz (Buchholz, 1970), and gentamicin-impregnated antibiotic beads were introduced as a local antibiotic carrier in the treatment of osteomyelitis by Klaus Klemm in the 1970s (Klemm, 1979). However, it was not until 1988 when two cases involving the use of gentamicin antibiotic beads in the treatment of pacemaker infections demonstrated the practical clinical application of the antibiotic bead pouch technique (Behrend, 1988). The most recent publication dedicated to this topic was in February 2023 (Patterson et al., 2023). The relatively rare indications and the heterogeneous patient population in which the technique is used may explain the limited number of publications on the topic. However, the success of NPWT, which has been an integral part of the daily routine of trauma surgery departments since the late 1990s, may also explain the low level of interest in the antibiotic bead pouch technique.

In 1989, the research group led by David Seligson in Louisville, Kentucky, first described the antibiotic bead pouch technique in a series of 21 Gustilo II and III tibia fractures. PMMA beads were loaded with tobramycin, opsite film provided temporary soft tissue coverage, and temporary fracture stabilization was performed. Film changes were required every 48–72 h. The advantage of a hematoma in the fracture area with high local antibiotic concentrations was supported by a positive culture rate of only 5 out of 86 in 46 procedures (Ostermann et al., 1989). The same research group conducted a comparative study immediately afterwards and demonstrated the positive effect of the technique on infection prevention in open Gustilo type IIIb and IIIc fractures requiring plastic coverage. With the local application of antibiotic bead chains in addition to systemic antibiotic prophylaxis, the osteomyelitis rate was significantly lower during the treatment course (14.3 % without vs. 2.4 % with local antibiotics) (Henry et al., 1990). Another publication, also from the Louisville group, reported a positive culture rate of 6.25 % (78 out of 1248) in a series of 204 fractures (Gustilo I–III) in 1993. On average, antibiotic bead pouches were changed twice (range of 1 to 7). Osteomyelitis developed in 0 % of Gustilo I fractures, 2.4 % of Gustilo II fractures, and 5.5 % of Gustilo III fractures (Henry et al., 1993). In a subgroup analysis with an expanded group size, the study group was also able to demonstrate lower infection rates with earlier soft tissue coverage after open fractures (without infection wound closure after a mean of 7.6 d, with infection after a mean of 17.9 d) (Ostermann et al., 1994). Particularly in severe soft tissue injuries in the setting of open fractures (Gustilo type IIIc), the antibiotic bead pouch technique demonstrated its positive effect (5 % infection with antibiotic bead pouch vs. 25 % without) (Seligson et al., 1994). Keating et al. (1996) demonstrated the positive results regarding infection reduction in a comparative analysis of Gustilo II–IIIb tibial fractures (4 % with antibiotic bead pouch vs. 16 % without antibiotic bead pouch). A first comparative study comparing NPWT and the antibiotic bead pouch was published in 2010. More surgical procedures were necessary when performing NPWT therapy, more MRSA infections occurred, and more unplanned surgical wound revisions were required. In addition, the authors demonstrated that in the United States, NPWT was USD 12 000 more expensive per patient compared to antibiotic bead pouch therapy (Warner et al., 2010). A study investigating the combined use of an antibiotic bead pouch with NPWT in a goat model was first published in 2012. It showed that the additional NPWT significantly reduced the efficiency of the antibiotic bead pouch in terms of antiseptic effect. Thus, after 2 d without additional NPWT, 6 times fewer bacteria were found in wounds previously contaminated with *Staphylococcus aureus* and surgically treated after 6 h (Stinner et al., 2012). Another experimental work in a goat model compared the combination therapy of NPWT with PMMA beads as an antibiotic carrier with a chitosan sponge as an antibiotic carrier. The use of an alternative chitosan sponge showed higher efficacy than PMMA, but it has not yet been established in clinical use analogous to the experimental setup (Rand and Wenke, 2017). Recently, Patterson et al. (2023) reported the results of using the antibiotic bead pouch for infection prevention in Gustilo IIIb open tibial shaft fractures and compared the outcome to NPWT treatment. The antibiotic bead pouch group had a lower risk for FRI requiring debridement or amputation than the NPWT group (Patterson et al., 2023).

## 7 Conclusion

The antibiotic bead pouch technique is a valuable, uncomplicated, and cost-effective solution for temporary soft tissue coverage. It allows for the delivery of high concentrations of antibiotics directly to the affected area, effectively reducing bacterial load and treating biofilm infections. This advantage is particularly relevant when compared to alternative treatments such as negative wound pressure therapy.

## Data Availability

The present review paper does not contain original data. The figures presented in this study include patient images and a drawing. The patient figures have been included with appropriate permissions and consent. Interested readers are welcome to utilize the figures included in this work, which are licensed under the Creative Commons Attribution 4.0 International License (CC BY 4.0). For additional details and associated data, researchers may contact the corresponding author.
